# Parent–child agreement in reporting somatic distress, gastrointestinal symptoms, mental health, and general health in girls with functional abdominal pain

**DOI:** 10.1007/s00431-025-06640-5

**Published:** 2025-11-22

**Authors:** Anna Duberg, Mats Eriksson, Anna Philipson

**Affiliations:** 1 Faculty of Medicine and Health, University Health Care Research Center, Örebro University, Universitetssjukhuset Örebro, S-huset, vån 2, SE-701 85, Örebro, Sweden; 2 Faculty of Medicine and Health, School of Health Sciences, Örebro University, Örebro, Sweden

**Keywords:** Functional abdominal pain disorders, Irritable bowel syndrome, Mental health symptoms, Parent–child agreement, General health, Somatic distress

## Abstract

To examine the inter-rater agreement between parent- and child-reported measures of somatic distress, gastrointestinal symptoms, mental health, and general health in girls aged 9–13 years with functional abdominal pain disorders (FAPDs). This secondary analysis used data from the Just in TIME randomized controlled trial, including girls aged 9–13 years with FAPDs. Both girls and their parents completed the Children’s Somatic Symptoms Inventory (gastrointestinal distress (CSSI-GI) and somatic distress (CSSI-nonGI) subscales) and answered questions about general health and mental health symptoms, measures at baseline and after 8 months. Agreement was assessed using intra-class correlation (ICC) and linear-weighted Cohen’s kappa. Data from 121 girls (mean age 10.6 years) and their parents were analysed. For CSSI-GI, mean scores were similar at baseline (girls 6.98, parents 7.27; ICC 0.84, *p* < .001) and follow-up (girls 4.25, parents 4.16; ICC 0.91, *p* < .001). For CSSI-nonGI, girls reported higher mean scores than parents at baseline (8.75 vs. 6.79; ICC 0.75, *p* < .001) and follow-up (7.14 vs. 5.43; ICC 0.78, *p* < .001). Agreement for general health was fair (kappa 0.31 at baseline, 0.41 at follow-up, both *p* < .001). For mental health symptoms, agreement ranged from slight to moderate (kappa 0.20–0.52 at baseline; 0.11–0.46 at follow-up, all significant).

*Conclusion*: Findings indicated good parent– and child agreement for gastrointestinal and general somatic symptoms, but lower agreement for mental health and general health.

*Clinical trial registration:* ClinicalTrials.gov, ID: NCT02920268, Name: Just in TIME–Intervention with Dance and Yoga for Girls with Recurrent Abdominal Pain, registered 29 September 2016.

**Table:** 

**What is Known:** • *Parent reports are commonly used in paediatric assessment, but it is recommended that children’s perspectives should be integrated in clinical practice and research.* • *Discrepancies between child and parent reports are frequent and may influence assessment accuracy and treatment planning.*
**What is New:** • *Parent–child agreement was higher for observable somatic symptoms, particularly gastrointestinal complaints, but lower for subjective experiences such as emotional states and perceived well-being.* • *Agreement for some items was higher at follow-up, suggesting potential gains in parental awareness or child–parent communication over time.*

**Supplementary Information:**

The online version contains supplementary material available at 10.1007/s00431-025-06640-5.

## Introduction

Functional abdominal pain disorders (FAPDs) are common in children and adolescents, particularly girls [1], and are often accompanied by somatic distress [2], school absenteeism [3], and psychological challenges [2, 4]. In the absence of definitive pathological biomarkers, clinical understanding of these conditions has largely relied on subjective reports. While parent reports are commonly used in paediatric assessments, it is essential to recognize children’s right to express their own experiences of health and illness. Aligned with international frameworks promoting child participation in healthcare [5], their subjective perspectives should be actively acknowledged and integrated into both clinical practice and research. However, discrepancies between child and parent reports are frequent [6, 7] and may influence assessment accuracy and treatment planning. It has been shown that parents underestimate pain-related functional disability [8] and anxiety [9] relative to children’s self-reports. Despite this, few studies have examined parent–child agreement in reports concerning paediatric FAPDs.

This study seeks to address this gap by exploring the agreement between girls 9 and 13 years old with functional abdominal pain (FAP) and their parents across multiple symptom domains—including somatic distress, gastrointestinal symptoms, mental health, and general health—before and after an 8-month dance and yoga intervention [10]. The selected items were designed to capture both physical symptoms, which are typically more observable, and psychological symptoms, which are often less visible and therefore more easily overlooked. Considering multiple perspectives is crucial when evaluating the complex symptomatology of FAPDs, particularly in multifaceted interventions.

The aim of this study was accordingly to examine the parent–child agreement in reporting somatic distress, gastrointestinal symptoms, and general and mental health in girls with FAPDs. Additionally, we aimed to examine whether the level of agreement differed at baseline and the 8-month follow-up.

## Materials and methods

### Source of data and study design

This study is based on data from a randomized controlled trial (RCT) including 121 girls aged 9–13 years with FAPDs, the Just in TIME (JiT) study [10, 11]. Participants and their legal guardians, here called parents, completed several instruments capturing both somatic distress and mental health. In the present study, we analysed data from instruments that both girls and their parents completed separately. This study was conducted in accordance with the Declaration of Helsinki, ethical approval was obtained from the Regional Ethical Review Board in Uppsala, Sweden (Dnr 2016/082 1–2). The article structure followed the Guidelines for Reporting Reliability and Agreement Studies (GRRAS) guidelines [12].

### Participants

Participants in the RCT were recruited from: outpatient clinics in the paediatric departments of four hospitals, school health services, and the general public (via media and website announcements) across two Swedish regions, Örebro and Västmanland. Additional recruitment occurred in Region Örebro through primary healthcare services and a counselling unit for children and adolescents. Eligible participants were girls aged 9–13 years diagnosed with FAP or irritable bowel syndrome (IBS) based on the Rome III criteria [13], who continued to experience pain following examination at a paediatric centre. Inclusion required a self-reported score of 4 or higher on the Faces Pain Scale–Revised (FPS-R) [14, 15] at least once during a week at baseline. Exclusion criteria were celiac disease, inflammatory bowel disease, difficulty following instructions, severe mental health issues, or current cognitive behavioural therapy (CBT). In addition to the girls, parents (at least one per participant) took part in all follow-ups. The full RCT design is described elsewhere [10]. Participation was voluntary, and parents provided informed consent.

### Procedure

The data analysed were collected at baseline and 8 months follow-up. Questionnaire sessions were conducted in an auditorium following school hours. Multiple members of the research team were present at each session to offer support and address any questions. Separate questionnaires were completed independently by the participating girls and one of their parents. For participants unable to attend the sessions, the questionnaires were distributed via postal mail.

### Measures

#### Somatic distress and gastrointestinal symptoms

Children’s Somatic Symptoms Inventory (CSSI)−24 was used to measure somatic distress and gastrointestinal symptoms [16, 17]. CSSI-24 is a measure available both as self-report and as a proxy, measuring the extent to which the child is affected by various non-specific somatic symptoms. The child and a parent indicate how much each symptom has bothered over the past 2 weeks, using a 5-point scale ranging from 0 (not at all) to 4 (a whole lot). Total scores are derived by summing the responses to all items, resulting in a possible range of 0 to 96 [16–18]. In this study, the total score, and the subscale for gastrointestinal (GI) and non-gastrointestinal (non-GI) symptoms have been used. The GI symptom subscale includes items referring to nausea, constipation, diarrhoea, stomach ache, vomiting, feeling bloated or gassy, and food making one sick; the remaining 17 items constitute the non-GI symptom subscale [17].

#### General health

General health was measured using SRH which encompasses aspects such as overall health and well-being and is commonly assessed using a single-item question: ‘How do you rate your general health? ‘For the purposes of this study, this item was adapted, as well, to allow for parents’ responses: ‘How do you rate your daughter’s general health?’. The response options for both versions of the single item range from 1 (very poor) to 5 (very good), with intermediate options being 2 (poor), 3 (neither good nor poor), and 4 (good). SRH is a stable and valid predictor of well-being in young people and is widely considered a reliable and comprehensive measure [19].

#### Mental health symptoms

Mental health questions originated from the regional general population survey Life & Health–Young People [20]. Participants rated the frequency with which they had experienced various emotional symptoms (i.e. stressed, nervous, anxious/worried, feeling down, energetic, irritated, happy, and relaxed/calm) over the previous 3 months on a 5-point scale (1 = never, 2 = infrequently, 3 = sometimes, 4 = frequently, and 5 = always).

#### Characteristics

Abdominal pain among children was measured with the Faces Pain Scale–Revised (FPS-R) [14, 15] in a pain diary for 1 week. The highest level during the week was categorized according to FPS-R scores on a 0–10 scale using Tsze’s 2018 criteria [21]: ≤ 2 = no pain, > 2–4 = mild pain, > 4–6 = moderate pain, and > 6 = severe pain. The girls’ age and country of birth were self-reported in their questionnaires at baseline. Diagnoses were extracted from medical records using the Rome III criteria [13]. Parents’ occupation and abdominal status were self-reported in their questionnaires.

### Statistical analyses

Data were analysed using IBM SPSS Statistics 29 for Windows (IBM Corp., Armonk, N.Y., USA). Sample characteristics were described using means with standard deviations or frequencies with percents.

The inter-rater agreement, meaning the level to which the assessments of two or more individuals (i.e. raters) match when using the same measure and assessing the same subject, was measured with the intra-class correlation coefficient (ICC) and weighted Cohen’s kappa (κ), according to the type of variable (i.e. continuous or categorical) [12]. These measures are the most robust measures of inter-rater agreement [22]. In line with methodological recommendations, linear weights for weighted Cohen’s kappa, the proportion of agreement, and category distribution (categorical variables) are presented [23]. Bland–Altman plots were created for continuous variables to evaluate the extent of disagreement and identify any systematic bias between parent and child responses.

ICCs range from 0 to 1, while weighted kappa values range between − 1 and 1. ICC values below 0.5 suggest low agreement among raters, whereas values of 0.5–0.75 indicate moderate agreement, 0.75–0.9 good agreement, and above 0.9 excellent agreement [24]. Weighted kappa values below 0 indicate no agreement, whereas values of 0.01–0.20 indicate no to slight agreement, 0.21–0.40 fair agreement, 0.41–0.60 moderate agreement, 0.61–0.80 substantial agreement, and 0.81–1.00 almost perfect agreement [25].

## Results

### Study sample

Baseline assessments included 121 child–parent pairs, of whom 101 completed the 8-month follow-up. All were girls with a mean age of 10.6 years (SD 1.37) at inclusion; most (61%) were diagnosed with FAP. Among the parents, a minority had abdominal pain problems (Table 1). At baseline, 61% of the girls reported moderate abdominal pain and 39% reported severe abdominal pain.

**Table 1:** Characteristics of the study sample

Children’s characteristics	
Age, mean (SD**)**	10.6 (1.366)
Diagnosis, *n* (%)	
* FAP*	74 (61.2)
* IBS*	47 (38.8)
Country of birth, *n* (%)	
* Sweden*	118 (97.5)
* Other country*	3 (2.5)
Parents’ characteristics	
Occupation, *n* (%)	
* Work or studies*	102 (85.7)
* Unemployed, sick leave, parental leave*	17 (14.3)
Abdominal diagnosis^a^ *n* (%)	
* Yes*	16 (13.3)
* No*	104 (86.7)
Abdominal symptoms^a^ *n* (%)	
* Yes*	39 (32.2)
* No*	82 (67.8)

^a^At least one parent. *FAP* functional abdominal pain, *IBS* irritable bowel syndrome

### Somatic distress and gastrointestinal symptoms

**Fig. 1:**
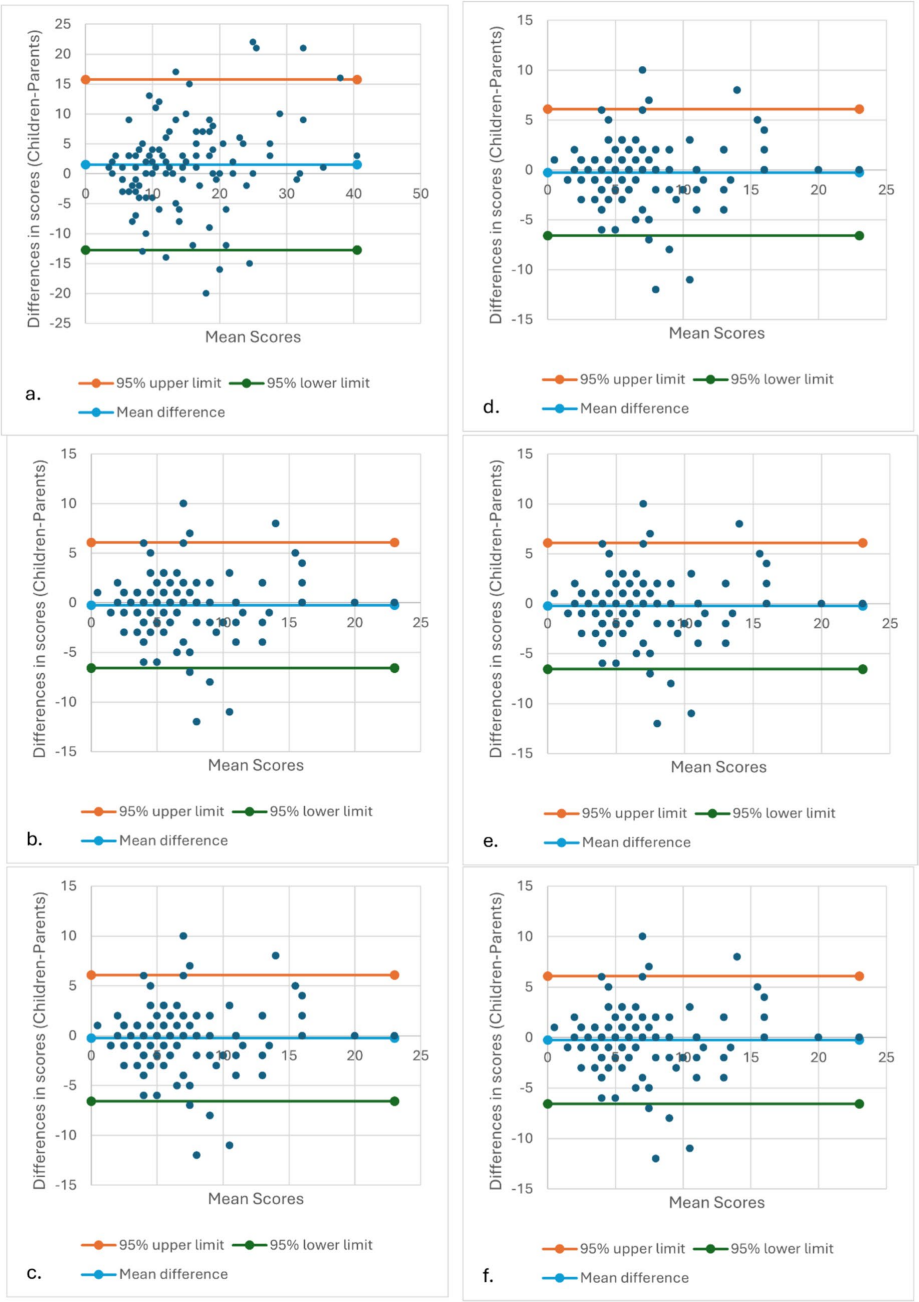
Bland–Altman plot comparing child and parental responses on the CSSI (total score, GI-subscale score, non-GI subscale score) at baseline and after 8 months. **a** Total score baseline. **b** GI-subscale score baseline. **c** Non-GI-subscale score baseline. **d** Total score 8-months. **e** GI-subscale score 8-months. **f** Non-GI-subscale score 8-months

At baseline, the ICC was 0.787 (confidence interval (CI) 0.688–0.854) for the CSSI total score, 0.840 (CI 0.769–0.889) for the GI subscale, and 0.752 (CI 0.620–0.836) for the non-GI subscale. Eight months later, the ICC was 0.827 (CI 0.729–0.889) for the CSSI total score, 0.906 (CI 0.858–0.937) for the GI subscale, and 0.778 (CI 0.639–0.860) for the non-GI subscale (Table 2). Agreement between the girls and their parents was also assessed using Bland–Altman plots (Fig. 1), which reveal differences between the girls’ and parents’ mean scores. These plots indicate that the girls’ scores, on average, were higher than their parents’ for the total score and the non-GI-subscale score at both time points, whereas the scores were more similar for the GI subscale at both time points. However, the 95% limits of agreement suggest that child–parent differences could vary at the individual level. The differences appeared to increase with higher scores, indicating some evidence of proportional bias.

**Table 2:** Child- vs. parent-report agreement, Children’s Somatic Symptoms Inventory, baseline and 8-month follow-up: mean values, intra-class correlation coefficient, and 95% confidence interval

	Children M (SD)	Parent M (SD)	ICC (*p*-value)	CI
*Baseline*				
CSSI-24	15.724 (9.529)	14.069 (8.059)	0.787 (< 0.001)	0.688–0.854
CSSI-24, GI subscale	6.983 (4.390)	7.271 (4.306)	0.840 (< 0.001)	0.769–0.889
CSSI-24, non-GI subscale	8.754 (6.508)	6.795 (5.049)	0.752 (< 0.001)	0.620–0.836
*Eight-month follow-up*				
CSSI-24	11.385 (8.261)	9.474 (7.086)	0.827 (< 0.001)	0.729–0.889
CSSI-24, GI subscale	4.250 (3.691)	4.165 (3.552)	0.906 (< 0.001)	0.858–0.937
CSSI-24, non-GI subscale	7.135 (5.759)	5.426 (4.916)	0.778 (< 0.001)	0.639–0.860

*CSSI* Children’s Somatic Symptoms Inventory, *GI* gastrointestinal, *ICC* intra-class correlation coefficient, *M* mean, *SD* standard deviation

The linear-weighted kappa values for CSSI items ranged from 0.234 (CI 0.100–0.368) to 0.764 (CI 0.591–0.936) at baseline and from 0.163 (CI 0.020–0.307) to 0.632 (CI 0.424–0.840) at the 8-month follow-up. Linear-weighted kappa values for GI-subscale items ranged from 0.355 (CI 0.232–0.478) to 0.764 (CI 0.591–0.936) at baseline and from 0.417 (CI 0.247–0.587) to 0.632 (CI 0.424–0.840) at the 8-month follow-up (Online resources 1 and 2).

Items displaying the best (i.e. substantial or perfect) agreement between the girls’ and their parents’ reports were as follows: at baseline—constipation, loose bowel movements/diarrhoea, vomiting, and food making one sick (all GI-subscale items); at 8 months—constipation and food making one sick. In addition, the item capturing pain in the lower back displayed high agreement at 8 months (Online resources 1 and 2).

Items displaying low (i.e. no, slight, or fair) agreement between the girls’ and their parents’ reports were as follows: at baseline—feeling low in energy/slowed down, numbness or tingling, heavy feelings in arms or legs, nausea or upset stomach, heart beating too fast, blurred vision, and pain in arms or legs; at 8-month follow-up—pain in heart or chest, sore muscles, trouble getting one’s breath, loss of voice, and weakness in parts of the body in addition to the baseline items (Online resources 1 and 2).

### General health

The agreement between the girls’ and their parents’ reports of general health was fair at baseline, with a linear-weighted kappa of 0.310 (CI, 0.159–0.462); 8 months later, the agreement had increased to a moderate level, with a linear-weighted kappa of 0.409 (CI, 0.267–0.552) (Table 3). Agreement proportions and category distributions are shown in Table 3 and Online resource 3.

**Table 3:** Child- vs. parent-report agreement, general health, measured with SRH, at baseline and 8-month follow-up: proportion of agreement, linear-weighted kappa, and 95% confidence interval

	Baseline			Eight-month follow-up		
	Proportion of agreement (SD)	Weighted Kappa (*p*-value), linear	CI	Proportion of agreement (SD)	Weighted Kappa (*p*-value), linear	CI
General health (SRH)	0.624 (0.486)	0.310 (< 0.001)	0.159–0.462	0.594 (0.494)	0.409 (< 0.001)	0.267–0.552

### Mental health

Moderate agreement was observed for the mental health items stressed, nervous, and feeling down at baseline, with linear-weighted kappa values of 0.430–0.518. Fair agreement was found for all other items, all with linear-weighted kappa values of 0.202–0.381 (Table 4).

**Table 4:** Child- vs. parent-report agreement, mental health, baseline and 8-month follow-up: proportion of agreement, linear-weighted kappa, and 95% confidence interval

	Baseline			Eight-month follow-up		
	Proportion of agreement (SD)	Weighted Kappa (*p*-value), linear	CI	Proportion of agreement (SD)	Weighted Kappa (*p*-value), linear	CI
Stressed	0.504 (0.502)	0.518 (< 0.001)	0.413–0.623	0.450 (0.500)	0.415 (< 0.001)	0.289–0.540
Nervous	0.530 (0.501)	0.430 (< 0.001)	0.302–0.558	0.495 (0.502)	0.437 (< 0.001)	0.310–0.565
Anxious/worried	0.379 (0.487)	0.381 (< 0.001)	0.276–0.486	0.485 (0.502)	0.111 (0.105)	–0.024–0.246
Feeling down	0.548 (0.500)	0.431 (< 0.001)	0.299–0.563	0.570 (0.498)	0.460 (< 0.001)	0.323–0.597
Energetic	0.504 (0.502)	0.319 (< 0.001)	0.190–0.449	0.545 (0.500)	0.331 (< 0.001)	0.180–0.482
Irritated	0.487 (0.502)	0.331 (< 0.001)	0.204–0.459	0.446 (0.500)	0.318 (< 0.001)	0.184–0.452
Happy	0.487 (0.502)	0.206 (< 0.001)	0.076–0.336	0.525 (0.502)	0.342 (< 0.001)	0.202–0.483
Relaxed/calm	0.453 (0.500)	0.202 (< 0.001)	0.073–0.331	0.390 (0.490)	0.165 (0.009)	0.025–0.305

By the 8-month follow-up, none of the mental health items had reached an agreement level higher than moderate. Agreement proportions and category distributions are shown in Table 4 and Online resource 4.

## Discussion

This study examined the agreement between girls aged 9–13 years with FAPDs and their parents in reporting somatic distress, gastrointestinal symptoms, mental health symptoms, and general health, both at two different time points. Overall, the highest agreement was found for observable and somatic symptoms, particularly gastrointestinal complaints, while agreement was notably lower for more subjective experiences, such as mental health and general health.

Our findings are in line with those reported in a systematic review and meta-analysis [22], which found generally low agreement between children’s self-reports and proxy ratings of health-related quality of life (HRQoL), particularly in domains involving less observable or more internal attributes such as psychosocial functioning. This pattern has also been demonstrated in previous parent–child research [26–30], underscoring how discrepancies in less visible somatic symptoms can pose a challenge for parents in identifying internal or emotionally driven experiences in their children.

In our study, moderate agreement was observed for stress, nervousness, and feeling down at both baseline and follow-up. All other mental health items displayed fair levels of agreement, likely reflecting the complex, internalized nature of some emotional states in young girls. This can also be related to the general tendency of children to seek greater independence as they grow older, implying that some girls in the study might not always have disclosed their feelings to their parents. Similar patterns of low parent–child agreement regarding emotional states have also been reported among children with chronic pain [28] and in diagnostic assessments of adolescent depression [31]. Interestingly, a recent study by Khanna et al. [32] involving generally healthy children found no significant differences in inter-rater agreement for HRQoL between children and parents, which may provide a basis for discussing how the child’s health status can influence communication with the parents. In children with FAPDs, findings regarding parent–child agreement vary. Lavigne et al. [33] reported generally low inter-rater agreement for children’s pain intensity, whereas Cunningham et al. [9] found that high inter-rater child–parent agreement of anxiety was associated with greater child-reported pain and disability, which may reflect the influence of parental perceptions on care-seeking.

Some items displayed good agreement at both measurement points. Substantial agreement was consistently observed for constipation and food making one sick. Additionally, agreement for the items feeling low in energy, nausea or upset stomach was higher at the 8-month follow-up, suggesting improved parental awareness or enhanced communication between parent and child regarding these symptoms. In contrast, some items, such as pain in arms and legs and weakness in parts of the body, displayed lower agreement at the 8-month follow-up. Symptoms such as numbness and blurred vision continued to display low agreement, likely due to their subjective or less observable nature. Taken together, the findings suggest that while observable somatic symptoms yield higher inter-rater agreement, emotional and internal experiences remain more difficult for parents to recognize in their daughters.

It is possible that the structured instruments and repeated measurements in the RCT may have created opportunities for parents and children to gradually develop a shared vocabulary for abdominal symptoms and related experiences. Although tentative, this interpretation is consistent with qualitative findings from the JiT study, in which the girls in the intervention group described growing confidence to open up and talk to their parents [34]. However, as this study was not designed to evaluate intervention effects, no causal conclusions can be drawn regarding the influence of the intervention on the agreement levels.

### Limitations and strengths

The study’s strengths include participants’ clinical diagnosis of FAPDs using validated ROME III criteria, the use of standardized assessment instruments and recommended statistical methods, and the use of data drawn from an RCT, all of which made it possible to analyse agreement at two time points. Of note, since study recruitment, Rome III criteria, have been updated to Rome IV, and FAPDs and IBS are now classified as Disorders of Gut–Brain Interaction (DGBIs). Some limitations of the study should also be acknowledged. One is that, although instructions specified that the same parent should complete the assessment at each time point, it cannot be confirmed that this was consistently the case. Relatedly, although no data were collected about which parent completed the questionnaires, the parent reports were primarily completed by the mothers, which may have influenced the observed agreement patterns. According to Poulain [30], an overrepresentation of mothers in a sample may yield higher agreement on psychosomatic complaints. 

### Implications and future directions

These findings highlight the importance of incorporating children’ perspectives in clinical assessments, particularly for paediatric populations with complex symptom profiles. While observable, physical symptoms tend to yield higher parent–child agreement, mental health symptoms and general health often rely on the child’s own internal experience and may be underreported or misinterpreted by caregivers. This underscores the value of including children’s self-reports, especially for emotional and subjective health dimensions, in both clinical and research settings. Children from about 6 years old are capable of reporting on their own health using questionnaires [35], however, to ensure reliability and validity, it is essential that such instruments be tailored to the child’s age, taking into account their cognitive and emotional development [36–38]. This calls for careful attention to the questionnaire’s design, format, and content to suit the specific target group [36]. Furthermore, it is generally recommended that responses be obtained directly from the children and adolescents themselves, rather than relying on proxy reporting [39]. Our findings align with previous research indicating that parent reports can facilitate evaluations but should not replace a child’s own reports [26, 40].

Future research should further investigate factors influencing parent–child agreement, including family communication, symptom visibility, and the broader psychosocial context. Longitudinal studies could also examine how agreement evolves over time in response to specific diagnoses and interventions. In addition, a study including more participants across different ages, areas and countries would increase the external validity of the results.

Clinically, the results support the use of multi-informant assessments while emphasizing the importance of child-centred evaluation tools to ensure a comprehensive understanding of children’s health and well-being. Incorporating standardized patient-reported outcome measures, e.g. the PROMIS® tool [41] may also facilitate the systematic inclusion of both children and parent perspectives, allowing clinicians to identify discrepancies in symptoms and support more individualized and comprehensive care.

## Conclusion

This study demonstrates that parent–child agreement in reporting symptoms among girls with functional abdominal pain disorders is highest for observable somatic and gastrointestinal complaints, while agreement remains lower for subjective domains such as mental health.

From a clinical perspective, these findings emphasize the importance of combining both children’s and parents’ perspectives in assessments, while ensuring that the children’s self-reports remain central, especially for internalizing symptoms. Supporting child-centred approaches could strengthen the accuracy and relevance of care in paediatric pain and mental health settings. Promoting children’s voices in health reporting aligns with ethical and rights-based approaches in paediatric care.

## Supplementary Information

Below is the link to the electronic supplementary material.

ESM 1 (PDF 171 KB)

ESM 2 (PDF 221 KB)

ESM 3 (PDF 123 KB)

ESM 4  (PDF 63.2 KB)

## Data Availability

The datasets generated and/or analyzed during the current study are not publicly available due current Swedish ethical legislation and the EU GDPR. However, data may be available from the corresponding author on reasonable request, provided that appropriate permissions are obtained from the relevant authorities.

## Abbreviations

CBT: Cognitive behavioural therapy
DGBI: Disorders of Gut–Brain Interaction
CSSI: Children’s Somatic Symptoms Inventory
FAP: Functional abdominal pain
FAPDs: Functional abdominal pain disorders
FPS-R: Faces Pain Scale–Revised
GI: Gastrointestinal
GRAAS: Guidelines for Reporting Reliability and Agreement Studies
IBS: Irritable bowel syndrome
ICC: Intra-class correlation coefficient
JiT: Just in TIME
PROMIS®: Patient-Reported Outcomes Measurement Information System
RCT: Randomized controlled trial
SRH: Self-rated health
