# Supplementary material for: Parent–child agreement in reporting somatic distress, gastrointestinal symptoms, mental health, and general health in girls with functional abdominal pain
Source: Eur J Pediatr. 2025 Nov 22;184(12):780. doi: 10.1007/s00431-025-06640-5 (PMC12640311; doi:10.1007/s00431-025-06640-5)
Supplement: Supplementary file 1 — (PDF 171 KB) [file 431_2025_6640_MOESM1_ESM.pdf]

Parent-child agreement in reporting somatic distress, gastrointestinal symptoms, mental health, and self-rated health in girls with functional abdominal pain

European Journal of Pediatrics

Anna Duberg[a], Mats Eriksson[b], Anna Philipson[a]*

[a] University Health Care Research Center, Faculty of Medicine and Health, Örebro University, Örebro, Sweden

[b] Faculty of Medicine and Health, School of Health Sciences, Örebro University, Örebro, Sweden

* anna.philipson@regionorebrolan.se

Distribution of the participants in the categories and linear-weighted kappa, CSSI, items, baseline

		Not at all	A little	Some	A lot	A whole lot	Linear-weighted Kappa (*p*-value)	CI
**Headaches**	Children, *n* (%)	16 (13.2)	50 41.3)	40 (33.1)	13 (10.7)	2 (1.7)	0.535 (<.001)	0.427–0.643
	Legal guardians, *n* (%)	20 (16.5)	47 (38.8)	42 (34.7)	8 (6.6)	4 (3.3)		
**Faintness or dizziness**	Children, *n* (%)	77 (63.6)	29 (24.0)	11 (9.1)	4 (3.3)	NA	0.483 (<.001)	0.347–0.619
	Legal guardians, *n* (%)	81 (66.9)	29 (24.0)	10 (8.3)	1 (0.8)	NA		
**Pain in the heart or chest**	Children, *n* (%)	84 69.4)	27 (22.3)	9 (7.4)	NA	1 (0.8)	0.468 (<.001)	0.306–0.631
	Legal guardians, *n* (%)	98 (82.4)	13 (10.9)	7 (5.9)	1 (0.8)	NA		
**Feeling low in energy or slowed down**	Children, *n* (%)	38 (31.7)	45 (37.5)	22 (18.3)	14 (11.7)	1 (0.8)	0.372 (<.001)	0.250–0.494
	Legal guardians, *n* (%)	41 (33.9)	47 (38.8)	24 (19.8)	7 (5.8)	2 (1.7)		
**Pain in lower back**	Children, *n* (%)	92 (76.7)	16 (13.3)	7 (5.8)	3 (2.5)	2 (1.7)	0.588 (<.001)	0.422–0.754
	Legal guardians, *n* (%)	98 (81.0)	13 (10.7)	6 (5.0)	2 (1.7)	2 (1.7)		
**Sore muscles**	Children, *n* (%)	72 (59.5)	30 (24.8)	15 (12.4)	3 (2.5)	1 (0.8)	0.437 (<.001)	0.301–0.572
	Legal guardians, *n* (%)	69 (57.0)	32 (26.4)	17 (14.0)	3 (2.5)	NA		
**Trouble getting your breath (when not exercising)**	Children, *n* (%)	97 (80.2)	17 (14.0)	5 (4.1)	1 (0.8)	1 (0.8)	0.552 (<.001)	0.376–0.727
	Legal guardians, *n* (%)	103 (85.1)	14 (11.6)	1 (0.8)	3 (2.5)	NA		
**Hot or cold spells**	Children, *n* (%)	60 (49.6)	38 (31.4)	16 (13.2)	4 (3.3)	3 (2.5)	0.437 (<.001)	0.299–0.575
	Legal guardians, *n* (%)	81 (66.9)	25 (20.7)	10 (8.3)	4 (3.3)	1 (0.8)		
**Numbness or tingling**	Children, *n* (%)	86 (71.1)	24 (19.8)	8 (6.6)	3 (2.5)	NA	0.234 (<.001)	0.100–0.368
	Legal guardians, *n* (%)	108 (90.0)	10 (8.3)	2 (1.7)	NA	NA		
**Weakness in parts of body**	Children, *n* (%)	75 (62.0)	36 (29.8)	9 (7.4)	NA	1 (0.8)	0.413 (<.001)	0.286–0.541
	Legal guardians, *n* (%)	92 (76.7)	23 (19.2)	5 (4.2)	NA	NA		
**Heavy feelings in arms or legs**	Children, *n* (%)	89 (74.2)	24 (20.0)	5 (4.2)	2 (1.7)	NA	0.371 (<.001)	0.203–0.539
	Legal guardians, *n* (%)	105 (87.5)	9 (7.5)	6 (5.0)	NA	NA		
**Nausea or upset stomach***	Children, *n* (%)	30 (24.8)	36 (29.8)	27 (22.3)	19 (15.7)	9 (7.4)	0.355 (<.001)	0.232–0.478
	Legal guardians, *n* (%)	24 (19.8)	29 (24.0)	36 (29.8)	24 (19.8)	8 (6.6)		
**Constipation***	Children, *n* (%)	64 (52.9)	31 (25.6)	16 (13.2)	4 (3.3)	6 (5.0)	0.660 (<.001)	0.552–0.768
	Legal guardians, *n* (%)	68 (56.7)	21 (17.5)	18 (15.0)	7 (5.8)	6 (5.0)		
**Loose bowel movements/diarrhoea***	Children, *n* (%)	77 (63.6)	26 (21.5)	12 (9.9)	4 (3.3)	2 (1.7)	0.608 (<.001)	0.475–0.742
	Legal guardians, *n* (%)	73 (61.3)	25 (21.0)	14 (11.8)	6 (5.0)	1 (0.8)		
**Pain in stomach or abdomen***	Children, *n* (%)	3 (2.5)	26 (21.8)	38 (31.9)	33 (27.7)	19 (16.0)	0.492 (<.001)	0.374–0.611
	Legal guardians, *n* (%)	2 (1.7)	24 (19.8)	40 (33.1)	39 (32.2)	16 (13.2)		
**Heart beating too fast (when**	Children, *n* (%)	96 (79.3)	15 (12.4)	10 (8.3)	NA	NA	0.340 (<.001)	0.157–0.523

**not exercising)**	Legal guardians, *n* (%)	100 (83.3)	16 (13.3)	4 (3.3)	NA	NA		
**Difficulty swallowing**	Children, *n* (%)	97 (80.2)	16 (13.2)	7 (5.8)	1 (0.8)	NA	0.410 (<.001)	0.244– .576
	Legal guardians, *n* (%)	110 (90.9)	9 (7.4)	1 (0.8)	1 (0.8)	NA		
**Loss of voice**	Children, *n* (%)	113 (93.4)	4 (3.3)	1 (0.8)	3 (2.5)	NA	0.599 (<.001)	0.272–0.925
	Legal guardians, *n* (%)	115 (95.0)	3 (2.5)	1 (0.8)	2 (1.7)	NA		
**Blurred vision (even with glasses on)**	Children, *n* (%)	103 (85.1)	12 (9.9)	4 (3.3)	2 (1.7)	NA	0.284 (<.001)	0.091–0.477
	Legal guardians, *n* (%)	109 (90.1)	11 (9.1)	NA	1 (0.8)	NA		
**Vomiting***	Children, *n* (%)	110 (90.9)	4 (3.3)	5 (4.1)	1 (0.8)	1 (0.8)	0.764 (<.001)	0.591–0.936
	Legal guardians, *n* (%)	109 (90.1)	6 (5.0)	4 (3.3)	2 (1.7)	NA		
**Bloating (gassy)***	Children, *n* (%)	46 (38.0)	38 (31.4)	20 (16.5)	13 (10.7)	4 (3.3)	0.519 (<.001)	0.394–0.644
	Legal guardians, *n* (%)	45 (37.2)	38 (31.4)	24 (19.8)	10 (8.3)	4 (3.3)		
**Food making one sick***	Children, *n* (%)	90 (74.4)	14 (11.6)	6 (5.0)	7 (5.8)	4 (3.3)	0.710 (<.001)	0.586–0.834
	Legal guardians, *n* (%)	90 (74.4)	10 (8.3)	15 (12.4)	4 (3.3)	2 (1.7)		
**Pain in knees, elbows, or other joints**	Children, *n* (%)	80 (66.1)	26 (21.5)	10 (8.3)	5 (4.1)	NA	0.479 (<.001)	0.353–0.606
	Legal guardians, *n* (%)	80 (66.1)	22 (18.2)	12 (9.9)	4 (3.3)	3 (2.5)		
**Pain in arms or legs**	Children, *n* (%)	75 (62.0)	33 (27.3)	8 (6.6)	4 (3.3)	1 (0.8)	0.375 (<.001)	0.233–0.517
	Legal guardians, *n* (%)	84 (69.4)	22 (18.2)	10 (8.3)	4 (3.3)	1 (0.8)		

* Included in the GI subscale
