# Supplementary material for: Parent–child agreement in reporting somatic distress, gastrointestinal symptoms, mental health, and general health in girls with functional abdominal pain
Source: Eur J Pediatr. 2025 Nov 22;184(12):780. doi: 10.1007/s00431-025-06640-5 (PMC12640311; doi:10.1007/s00431-025-06640-5)
Supplement: Supplementary file 2 — (PDF 221 KB) [file 431_2025_6640_MOESM2_ESM.pdf]

Parent–child agreement in reporting somatic distress, gastrointestinal symptoms, mental health, and self-rated health in girls with functional abdominal pain

European Journal of Pediatrics

Anna Duberg[a], Mats Eriksson[b], Anna Philipson[a]*

[a] University Health Care Research Center, Faculty of Medicine and Health, Örebro University, Örebro, Sweden

[b] Faculty of Medicine and Health, School of Health Sciences, Örebro University, Örebro, Sweden

* anna.philipson@regionorebrolan.se

Distribution of the participants in the categories and linear-weighted kappa, CSSI, items, eight-month follow-up

		Not at all	A little	Some	A lot	A whole lot	Linear-weighted Kappa (*p*-value)	CI
**Headaches**	Children, *n* (%)	25 (24.8)	44 (43.7)	21 (20.8)	10 (9.9)	1 (1.0)	0.525 (<.001)	0.402–0.649
	Legal guardians, *n* (%)	24 (23.8)	48 (47.5)	21 (20.8)	7 (6.9)	1 (1.0)		
**Faintness or dizziness**	Children, *n* (%)	71 (70.3)	21 (20.8)	7 (6.9)	2 (2.0)	NA	0.465 (<.001)	0.312–0.618
	Legal guardians, *n* (%)	75 (74.2)	25 (24.8)	1 (1.0)	NA	NA		
**Pain in the heart or chest**	Children, *n* (%)	78 (78.8)	15 (15.2)	5 (5.1)	1 (1.0)	NA	0.339 (<.001)	0.124–0.555
	Legal guardians, *n* (%)	89 (88.1)	8 (7.9)	4 (4.0)	NA	NA		
**Feeling low in energy or slowed down**	Children, *n* (%)	34 (33.7)	41 (40.6)	17 (16.8)	8 (7.9)	1 (1.0)	0.521 (<.001)	0.396–0.647
	Legal guardians, *n* (%)	46 (45.5)	33 (32.7)	19 (18.8)	3 (3.0)	NA		
**Pain in lower back**	Children, *n* (%)	86 (86.0)	7 (7.0)	4 (4.0)	1 (1.0)	2 (2.0)	0.629 (<.001)	0.406–0.853
	Legal guardians, *n* (%)	87 (86.1)	7 (6.9)	4 (4.0)	2 (2.0)	1 (1.0)		
**Sore muscles**	Children, *n* (%)	65 (64.4)	24 (23.8)	8 (7.9)	4 (4.0)	NA	0.273 (<.001)	0.104–0.443
	Legal guardians, *n* (%)	64 (63.4)	20 (19.8)	15 (14.9)	1 (1.0)	1 (1.0)		
**Trouble getting one's breath (when not exercising)**	Children, *n* (%)	77 (76.2)	17 (16.8)	6 (5.9)	1 (1.0)	NA	0.371 (<.001)	0.186–0.555
	Legal guardians, *n* (%)	64 (63.4)	20 (19.8)	15 (14.9)	1 (1.0)	1 (1.0)		
**Hot or cold spells**	Children, *n* (%)	71 (71.0)	16 (16.0)	8 (8.0)	3 (3.0)	2 (2.0)	0.471 (<.001)	0.269–0.672
	Legal guardians, *n* (%)	82 (81.2)	9 (8.9)	7 (6.9)	2 (2.0)	1 (1.0)		
**Numbness or tingling**	Children, *n* (%)	65 (64.4)	28 (27.7)	4 (4.0)	3 (3.0)	1 (1.0)	0.163 (0.013)	0.020–0.307
	Legal guardians, *n* (%)	86 (85.1)	13 (12.9)	2 (2.0)	NA	NA		
**Weakness in parts of body**	Children, *n* (%)	65 (64.4)	25 (24.8)	9 (8.9)	2 (2.0)	NA	0.279 (0.001)	0.117–0.441
	Legal guardians, *n* (%)	86 (85.1)	14 (13.9)	1 (1.0)	NA	NA		
**Heavy feelings in arms or legs**	Children, *n* (%)	84 (83.2)	15 (14.9)	2 (2.9)	NA	NA	0.323 (<.001)	0.094–0.552
	Legal guardians, *n* (%)	91 (90.1)	9 (8.9)	1 (1.0)	NA	NA		
**Nausea or upset stomach***	Children, *n* (%)	49 (48.5)	31 (30.7)	13 (12.9)	3 (3.0)	5 (5.0)	0.509 (<.001)	0.382–0.635
	Legal guardians, *n* (%)	38 (37.6)	33 (32.7)	21 (20.8)	6 (5.9)	3 (3.0)		
**Constipation***	Children, *n* (%)	70 (70.0)	15 (15.0)	5 (5.0)	8 (8.0)	2 (2.0)	0.625 (<.001)	0.494–0.756
	Legal guardians, *n* (%)	68 (68.0)	19 (19.0)	7 (7.0)	4 (4.0)	2 (2.0)		
**Loose bowel movements/diarrhoea***	Children, *n* (%)	74 (74.0)	16 (16.0)	6 (6.0)	2 (2.0)	2 (2.0)	0.559 (<.001)	0.405–0.714
	Legal guardians, *n* (%)	71 (71.0)	17 (17.0)	9 (9.0)	3 (3.0)	NA		
**Pain in stomach or abdomen***	Children, *n* (%)	22 (22.0)	33 (33.0)	21 (21.0)	18 (18.0)	6 (6.0)	0.544 (<.001)	0.428–0.660
	Legal guardians, *n* (%)	20 (19.8)	39 (38.6)	23 (22.8)	16 (15.8)	3 (3.0)		
**Heart beating too fast (when**	Children, *n* (%)	88 (88.0)	8 (8.0)	3 (3.0)	1 (1.0)	NA	0.333 (<.001)	0.062–0.605

**not exercising)**	Legal guardians, *n* (%)	92 (91.1)	5 (5.0)	3 (3.0)	1 (1.0)	NA		
**Difficulty swallowing**	Children, *n* (%)	86 (86.0)	8 (8.0)	3 (3.0)	NA	3 (3.0)	0.414 (<.001)	0.240–0.589
	Legal guardians, *n* (%)	91 (90.1)	7 (6.9)	3 (3.0)	NA	NA		
**Loss of voice**	Children, *n* (%)	92 (92.0)	6 (6.0)	1 (1.0)	NA	1 (1.0)	0.203 (0.004)	–0.124–0.531
	Legal guardians, *n* (%)	97 (96.0)	3 (3.0)	1 (1.0)	NA	NA		
**Blurred vision (even with glasses on)**	Children, *n* (%)	83 (83.0)	13 (13.0)	4 (4.0)	NA	NA	0.338 (<.001)	0.073–0.604
	Legal guardians, *n* (%)	93 (92.1)	7 (6.9)	1 (1.0)	NA	NA		
**Vomiting***	Children, *n* (%)	91 (91.0)	5 (5.0)	4 (4.0)	NA	NA	0.503 (<.001)	0.209–0.796
	Legal guardians, *n* (%)	94 (95.9)	2 (2.0)	2 (2.0)	NA	NA		
**Bloating (gassy)***	Children, *n* (%)	64 (65.3)	25 (25.5)	6 (6.1)	3 (3.1)	NA	0.417 (<.001)	0.247–0.587
	Legal guardians, *n* (%)	63 (63.0)	27 (27.0)	9 (9.0)	NA	1 (1.0)		
**Food making one sick***	Children, *n* (%)	86 (86.0)	10 (10.0)	2 (2.0)	2 (2.0)	NA	0.632 (<.001)	0.424–0.840
	Legal guardians, *n* (%)	90 (89.1)	8 (7.9)	1 (1.0)	2 (2.0)	NA		
**Pain in knees, elbows, or other joints**	Children, *n* (%)	70 (70.7)	21 (21.2)	4 (4.0)	4 (4.0)	NA	0.408 (<.001)	0.225–0.592
	Legal guardians, *n* (%)	72 (71.3)	15 (14.9)	10 (9.9)	3 (3.0)	1 (1.0)		
**Pain in arms or legs**	Children, *n* (%)	75 (75.0)	17 (17.0)	6 (6.0)	2 (2.0)	NA	0.287 (<.001)	0.101–0.473
	Legal guardians, *n* (%)	74 (73.3)	18 (17.8)	6 (5.9)	3 (3.0)	NA		

* Included in the GI subscale
