# Supplementary material for: Parent–child agreement in reporting somatic distress, gastrointestinal symptoms, mental health, and general health in girls with functional abdominal pain
Source: Eur J Pediatr. 2025 Nov 22;184(12):780. doi: 10.1007/s00431-025-06640-5 (PMC12640311; doi:10.1007/s00431-025-06640-5)
Supplement: Supplementary file 3 — (PDF 123 KB) [file 431_2025_6640_MOESM3_ESM.pdf]

Parent–child agreement in reporting somatic distress, gastrointestinal symptoms, mental health, and self-rated health in girls with functional abdominal pain

European Journal of Pediatrics

Anna Duberg[a], Mats Eriksson[b], Anna Philipson[a]*

[a] University Health Care Research Center, Faculty of Medicine and Health, Örebro University, Örebro, Sweden

[b] Faculty of Medicine and Health, School of Health Sciences, Örebro University, Örebro, Sweden

* anna.philipson@regionorebrolan.se

Distribution of the participants in the categories, mental health

		Never	Rarely	Sometimes	Often	Always
Baseline						
**Stressed, *n* (%)**	Children, *n* (%)	18 (14.9)	31 (25.6)	44 (36.4)	23 (19.0)	5 (4.1)
	Legal guardians, *n* (%)	15 (13.0)	26 (22.6)	45 (39.1)	27 (23.5)	2 (1.7)
**Nervous, *n* (%)**	Children, *n* (%)	22 (18.3)	33 (27.5)	47 (38.8)	16 (13.3)	2 (1.7)
	Legal guardians, *n* (%)	15 (13.0)	26 (22.6)	45 (39.1)	27 (23.5)	2 (1.7)
**Anxious/worried, *n* (%)**	Children, *n* (%)	36 (30.0)	39 (32.5)	26 (21.7)	16 (13.3)	3 (2.5)
	Legal guardians, *n* (%)	19 (16.2)	21 (17.9)	44 (37.6)	30 (25.6)	3 (2.6)
**Feeling down, *n* (%)**	Children, *n* (%)	43 (36.1)	40 (33.6)	29 (24.4)	7 (5.9)	NA
	Legal guardians, *n* (%)	34 (29.1)	39 (33.3)	32 (27.4)	11 (9.4)	1 (0.9)
**Energetic, *n* (%)**	Children, *n* (%)	4 (3.3)	16 (13.3)	40 (33.3)	52 (43.3)	8 (6.7)
	Legal guardians, *n* (%)	1 (0.8)	10 (8.5)	39 (33.1)	59 (50.0)	9 (7.6)
**Irritated, *n* (%)**	Children, *n* (%)	13 (10.7)	31 (25.6)	49 (40.5)	26 (21.5)	2 (1.7)
	Legal guardians, *n* (%)	6 (5.0)	26 (21.8)	49 (41.2)	36 (30.3)	2 (1.7)
**Happy, *n* (%)**	Children, *n* (%)	3 (2.5)	8 (6.7)	32 (26.7)	65 (54.2)	12 (10.0)
	Legal guardians, *n* (%)	NA	6 (5.4)	39 (35.1)	66 (59.5)	NA
**Relaxed/calm, *n* (%)**	Children, *n* (%)	5 (4.2)	23 (19.2)	52 (43.3)	36 (30.0)	4 (3.3)
	Legal guardians, *n* (%)	1 (0.8)	15 (12.7)	52 (44.1)	50 (42.4)	NA
Eight-month follow-up						
**Stressed, *n* (%)**	Children, *n* (%)	23 (23.0)	34 (34.0)	27 (27.0)	14 (14.0)	2 (2.0)
	Legal guardians, *n* (%)	18 (18.0)	26 (26.0)	37 (37.0)	19 (19.0)	NA
**Nervous, *n* (%)**	Children, *n* (%)	26 (25.7)	35 (34.7)	30 (29.7)	7 (6.9)	3 (3.0)
	Legal guardians, *n* (%)	32 (32.0)	28 (28.0)	26 (26.0)	14 (14.0)	NA
**Anxious/worried, *n* (%)**	Children, *n* (%)	45 (45.0)	29 (29.0)	17 (17.0)	7 (7.0)	2 (2.0)
	Legal guardians, *n* (%)	27 (27.8)	27 (27.8)	29 (30.0)	14 (14.4)	NA
**Feeling down, *n* (%)**	Children, *n* (%)	38 (38.0)	35 (35.0)	22 (22.0)	4 (4.0)	1 (1.0)
	Legal guardians, *n* (%)	39 (39.0)	36 (36.0)	17 (17.0)	8 (8.0)	NA
**Energetic, *n* (%)**	Children, *n* (%)	4 (4.0)	14 (13.9)	31 (30.7)	45 (44.6)	7 (6.9)
	Legal guardians, *n* (%)	2 (2.0)	3 (3.0)	31 (31.0)	58 (58.0)	6 (6.0)
**Irritated, *n* (%)**	Children, *n* (%)	16 (15.8)	36 (35.6)	30 (29.7)	16 (15.8)	3 (3.0)
	Legal guardians, *n* (%)	8 (7.9)	26 (25.7)	46 (45.5)	20 (19.8)	1 (0.9)
**Happy, *n* (%)**	Children, *n* (%)	NA	18 (17.8)	23 (22.8)	43 (42.6)	17 (16.8)
	Legal guardians, *n* (%)	1 (1.0)	7 (7.0)	31 (31.0)	54 (54.0)	7 (7.0)
**Relaxed/calm, *n* (%)**	Children, *n* (%)	5 (5.0)	17 (16.8)	40 (39.6)	33 (32.7)	6 (5.9)
	Legal guardians, *n* (%)	3 (3.0)	9 (9.0)	30 (30.0)	55 (55.0)	3 (3.0)
