# Supplementary material for: Parent–child agreement in reporting somatic distress, gastrointestinal symptoms, mental health, and general health in girls with functional abdominal pain
Source: Eur J Pediatr. 2025 Nov 22;184(12):780. doi: 10.1007/s00431-025-06640-5 (PMC12640311; doi:10.1007/s00431-025-06640-5)
Supplement: Supplementary file 4 — (PDF 63.2 KB) [file 431_2025_6640_MOESM4_ESM.pdf]

Parent–child agreement in reporting somatic distress, gastrointestinal symptoms, mental health, and self-rated health in girls with functional abdominal pain

European Journal of Pediatrics

Anna Duberg[a], Mats Eriksson[b], Anna Philipson[a]*

[a] University Health Care Research Center, Faculty of Medicine and Health, Örebro University, Örebro, Sweden

[b] Faculty of Medicine and Health, School of Health Sciences, Örebro University, Örebro, Sweden

* anna.philipson@regionorebrolan.se

Distribution of the participants in the categories, self-rated health

	**Very good**	**Good**	**Neither good nor poor**	**Poor**	**Very poor**
Baseline					
**Children,**[a] ***n* (%)**	11 (9.1)	73 (60.3)	35 (28.9)	2 (1.7)	NA
**Legal guardians,**[b] ***n* (%)**	14 (12.0)	74 (63.2)	27 (23.1)	2 (1.7)	NA
Eight-month follow-up					
**Children,**[c] ***n* (%)**	22 (21.8)	60 (59.4)	16 (15.8)	1 (<0.1)	2 (1.7)
**Legal guardians,**[d] ***n* (%)**	16 (15.8)	68 (67.3)	13 (12.9)	4 (4.0)	NA
